# RNA sequencing-based identification of the regulatory mechanism of microRNAs, transcription factors, and corresponding target genes involved in vascular dementia

**DOI:** 10.3389/fnins.2022.917489

**Published:** 2022-09-20

**Authors:** Kaiyue Zhao, Li Zeng, Zhongdi Cai, Mimin Liu, Ting Sun, Zhuorong Li, Rui Liu

**Affiliations:** Institute of Medicinal Biotechnology, Chinese Academy of Medical Sciences and Peking Union Medical College, Beijing, China

**Keywords:** microRNA, RNA sequencing, regulatory network, transcription factor, vascular dementia

## Abstract

Vascular dementia (VaD) is the second most common form of dementia with uncertain mechanisms and no effective treatments. microRNAs (miRNAs) and transcription factors (TFs) are considered regulatory factors of genes involved in many diseases. Therefore, this work investigated the aberrantly expressed miRNAs, TFs, corresponding target genes, and their co-regulatory networks in the cortex of rats with bilateral common carotid artery occlusion (2VO) to uncover the potential mechanism and biomarkers of VaD. Differentially expressed genes (DEGs), miRNAs (DEMs), and TFs (DETFs) were identified using RNA sequencing, and their interaction networks were constructed using Cytoscape. The results showed that rats with 2VO had declined cognitive abilities and neuronal loss in the cortex than sham rats. DEGs, DEMs, and DETFs were discriminated between rats with 2VO and sham rats in the cortex, as shown by the 13 aberrantly expressed miRNAs, 805 mRNAs, and 63 TFs. The miRNA-TF-target gene network was constructed, showing 523 nodes and 7237 edges. Five miRNAs (miR-5132-5p, miR-764-3p, miR-223-3p, miR-145-5p, and miR-122-5p), ten TFs (*Mxi1*, *Nfatc4*, *Rxrg*, *Zfp523*, *Foxj2*, *Nkx6*-1, *Klf4*, *Klf5*, *Csrnp1*, and *Prdm6*), and seven target genes (*Serpine1*, *Nedd4l*, *Pxn*, *Col1a1*, *Plec*, *Trip12*, and *Tpm1*) were chosen as the significant nodes to construct feed-forward loops (FFLs). Gene Ontology and pathway enrichment analysis revealed that these miRNA and TF-associated genes are mostly involved in the PI3K/Akt, neuroactive ligand–receptor interaction, calcium signaling, and Wnt signaling pathways, along with central locations around the cell membrane. They exert functions such as growth factor binding, integrin binding, and extracellular matrix structural constituent, with representative biological processes like vasculature development, cell–substrate adhesion, cellular response to growth factor stimulus, and synaptic transmission. Furthermore, the expression of three miRNAs (miR-145-5p, miR-122-5p, and miR-5132-5p), six TFs (*Csrnp1*, *Klf4*, *Nfatc4*, *Rxrg*, *Foxj2*, and *Klf5*), and five mRNAs (*Serpine1*, *Plec*, *Nedd4l*, *Trip12*, and *Tpm1*) were significantly changed in rats with VaD, in line with the outcome of RNA sequencing. In the potential FFL, miR-145-5p directly bound *Csrnp1* and decreased its mRNA expression. These results might help the understanding of the underlying regulatory mechanisms of miRNA-TF-genes, providing potential therapeutic targets in VaD.

## Introduction

Vascular dementia (VaD) is the second cause of dementia after Alzheimer’s disease (AD), characterized by brain impairments due to inadequate blood perfusion. The patients suffering from VaD undergo cognitive decline and executive dysfunction. Stroke and infarct of cerebral vessels are associated with VaD, and hypertension, atherosclerosis, cardiac disease, and diabetes are additional risks for developing VaD (Venkat et al., 2015). The acute or chronic deterioration of cerebral vessels causes oxygen deprivation following the energy strike of neurons, leading to oxygen stress reaction, microglial and astroglia activation, mitochondrial injuries, loss of synaptic function, neuron apoptosis, or necrosis (Sun, 2018). The pathology of VaD is complex and unclear due to the heterogeneity of vascular diseases. Neuroimaging assessment and cognitive evaluation are the current methods in the diagnosis of VaD, but both have shortcomings, thus weakening the accuracy of the diagnosis (van der Flier et al., 2018). Besides, no officially therapeutic drugs are approved for VaD management, and drugs used for improving cognitive impairments mainly for AD are tentatively used in VaD, such as cholinesterase inhibitors and memantine (O’Brien and Thomas, 2015). Thus, the investigation of the VaD mechanism of action is urgently needed to facilitate the diagnosis or intervention of this disease.

MicroRNAs (miRNAs) are short non-coding RNAs repressing mRNA expression post-transcriptionally through the induction of degradation or translational repression of the target gene. Dysregulated miRNAs act as potential biomarkers in the diagnosis and prognosis of several diseases (Ragusa et al., 2016; Jiang et al., 2022). They also serve as targets for the intervention on the symptoms of the disease (Chen et al., 2017; Yao et al., 2017; Zeng et al., 2021a; Sun et al., 2022). Multiple miRNAs such as miR-145, novel miRNA PC-5P-12969, and miR-134-5p are reported as dysregulated in the process of neurological injuries induced by ischemia and are involved in the cognitive impairment of VaD (Dharap et al., 2009; Liu et al., 2019; Vijayan et al., 2019).

Aberrantly expressed miRNAs are closely associated with transcription factors (TFs). In turn, TFs are involved in the control of signaling pathways with miRNAs through the activation or suppression of the transcription of target genes by binding to the promoter and enhancer sequences (Spitz and Furlong, 2012; Inukai et al., 2017; Zhang et al., 2018). Furthermore, TFs and miRNAs interact with each other through complex molecular regulatory mechanisms (Zhang et al., 2015), which play a crucial part in the pathogenesis of VaD (Saggu et al., 2016; Han et al., 2020; Yang and Zhang, 2021). For example, the nuclear factor erythroid 2-related factor 2 (*Nrf2*), considered as the essential transcription factor for antioxidant responses, controls the transcription of a series of anti-oxidative and anti-inflammatory genes (Li et al., 2020a; Saha et al., 2020); as the functional target of miR-153 and miR-144, *Nrf2* ameliorates oxidative stress and inhibits the apoptotic signaling pathways (Chu et al., 2019; Zhu et al., 2019). Forkhead box P2 (*Foxp2*) is another transcription factor involved in early VaD and improves cognitive impairment in rats with VaD through the upregulation of synaptic proteins through the miR-134-5p/*Foxp2*/*Syn1* axis (Liu et al., 2019). The protective effect of acupuncture treatment in a rodent model of VaD may be related to the suppression of the miR-93-induced TLR4/MyD88/NF-κB pathway (Wang et al., 2020). However, the interrelationship of miRNAs, TFs, and target genes in VaD has not been fully elucidated.

**FIGURE 1:**
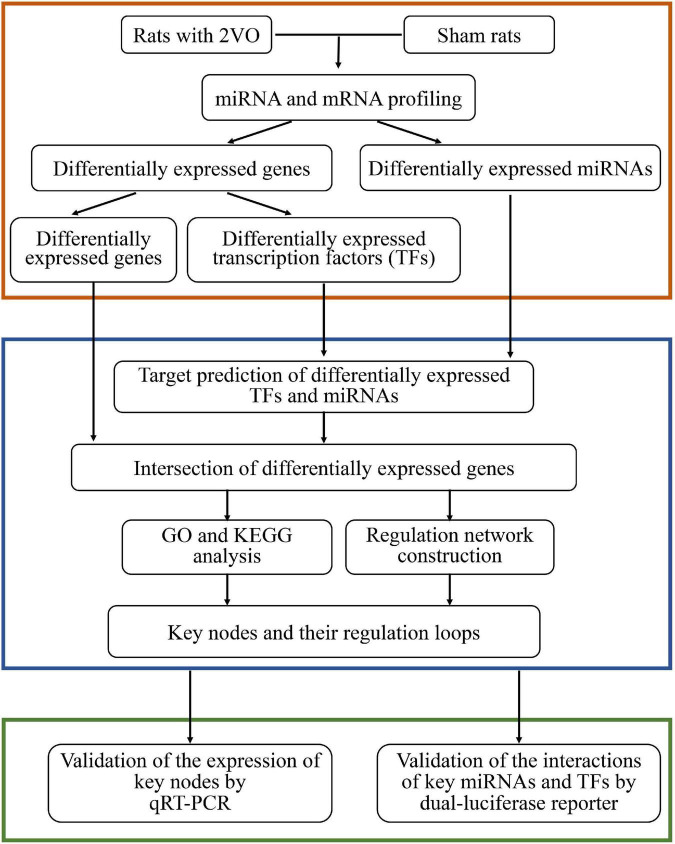
Workflow of the present study.

In the present study, rats subjected to bilateral vessel occlusion (2VO), a widely accepted model induced by chronic blood hypoperfusion (Weinstock and Shoham, 2004), were used to mimic the pathology of VaD. Dysregulated miRNAs and mRNAs were obtained from the transcriptome sequencing of rats with VaD and sham rats. The regulation of differentially expressed miRNAs (DEMs), TFs (DETFs), and target genes (DEGs) was analyzed, followed by the construction of an integrative co-regulatory gene expression network. The expression of key DEMs, DETFs, and DEGs and their potential interaction were further verified to provide new insights into the mechanism of VaD. The workflow of the present study is presented in Figure 1.

## Materials and methods

### Animals

**FIGURE 2:**
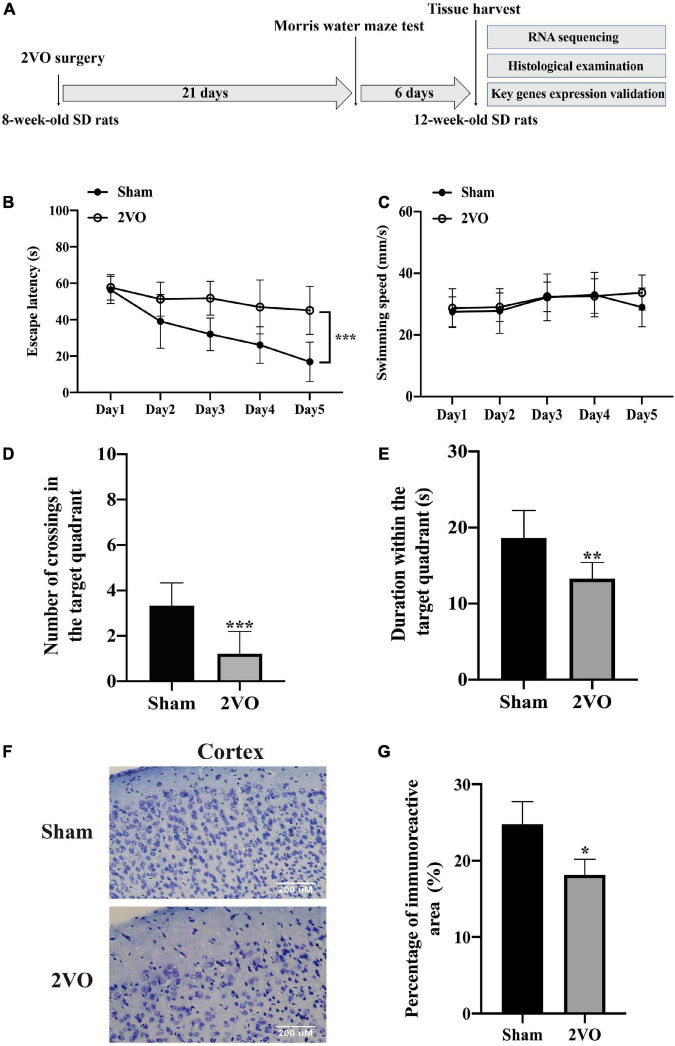
Cognitive decline and neuronal injury in rats with 2VO compared with sham rats. **(A)** Timeline of the *in vivo* experiment. 8-week-old SD rats were subjected to the bilateral common carotid artery occlusion, followed by the cognitive impairment test after 3 weeks. The cerebral cortices were finally collected from the 12-week-old SD rats. 2VO: bilateral common carotid artery occlusion. **(B)** Escape latency of rats in the navigation test for five consecutive days (*n* = 9). **(C)** Swimming speed of rats during the navigation test (*n* = 9). **(D)** Number of crossings of rats to find platforms previously located in the probe test (*n* = 9). **(E)** Duration time in the target quadrant in the probe test (*n* = 9). **(F)** Representative images of Nissl staining in the cortex of 2VO and sham rats. **(G)** Quantification of Nissl staining (*n* = 3). Results are presented as mean ± SD. *P < 0.05, **P < 0.01, ***P < 0.001 *vs*. sham.

Male Sprague–Dawley (SD) rats (270 ± 10 g in weight, 7-week-old) were purchased from the Zhishan Healthcare Research Institute (Beijing, China) and maintained under the specified pathogen-free conditions with regular rat chow and water *ad libitum*. After 1 week of adaptively feeding, they were used for the construction of the *in vivo* VaD model as previously reported (Niu et al., 2019; Guo et al., 2020; Mao et al., 2020). All the experimental procedures were approved by the Committee of the Institute of Medicinal Biotechnology, China (No. IMB-201807-D8-03). Eighteen rats were randomly assigned to two groups and subjected to surgery. Nine rats in each group were subjected to the Morris water maze (MWM) test and then randomly divided into three equal parts to collect the cerebral cortex for the RNA sequencing analysis, histological examination by Nissl staining, and gene validation analysis. The overall experimental protocol is shown in Figure 2A.

### Surgical procedure for bilateral vessel occlusion in rats

The VaD model was induced in rats by the permanent occlusion of the bilateral common carotid artery, as described in our previous work (Jiang et al., 2021). Briefly, rats were anesthetized with an intraperitoneal injection of 2% sodium pentobarbital (30 mg/kg). The bilateral common carotid arteries were gently separated and permanently ligated with a 5–0 silk thread. Rats in the sham group underwent the same procedure except for the arterial ligation.

### Spatial cognitive evaluation

The MWM test was performed for the spatial cognitive evaluation on the 21st day after the occlusion surgery, as previously described (Liu et al., 2018; Jiang et al., 2021). The test was represented by the navigation trial and the probe test. The navigation test lasted for five consecutive days. The rats experienced four pieces of training for the ability to search the underwater platform each day. The time the rats spent searching the platform was recorded as the escape latency. The probe test was performed 24 h after the navigation test, in which the hidden platform was removed, and the time the rats spent in the target quadrant and the frequency crossing the platform were recorded.

### Histological examination

After the MWM test, routine Nissl staining was performed to evaluate the neuronal injury induced by 2VO. Briefly, rats were anesthetized with sodium pentobarbital and transcardially perfused with saline and 4% paraformaldehyde (PFA). The isolated whole brains were fixed in 4% PFA for 24 h at 4°C and then cryoprotected in ascending sucrose series gradient in phosphate-buffered saline. The slide-mounted brain sections (4 μm thick) were differentiated in 95% ethyl alcohol, rinsed in 75% ethyl alcohol and distilled water, and stained with 0.1% cresyl violet solution (Sigma, St. Louis, MI, United States). After a rinse in distilled water and dehydration in 95 and 100% ethyl alcohol, the sections were finally cleared in xylene for 2–3 min and mounted with Permount (Sigma, St. Louis, MI, United States). Images were acquired using a fluorescence microscope (Olympus, Tokyo, Japan).

### RNA isolation, library preparation, sequencing, and data processing

The protocols of total RNA isolation and sequencing of miRNA or mRNA were the same as described in our previous work (Zeng et al., 2021b). Briefly, total RNA was extracted using the Total RNA Extractor (Trizol) kit (B511311, Sangon, China) according to the manufacturer’s protocol, and the sample quality was evaluated using a 1.0% agarose gel for RNA integrity and genomic pollution. An amount of 2 μg RNA per sample was used as input material for the RNA sample preparation. Next, six mRNA sequencing libraries were constructed from six total RNA samples using VAHTSTM mRNA-seq V2 Library Prep Kit for Illumina^®^ (NR601-02, Vazyme, China). Moreover, six sequencing libraries of miRNA were prepared from the same six total RNA samples using TruSeq Small RNA Sample Prep Kits (RS-200-0024, Illumina, United States), following the manufacturer’s recommendations. The library quality was assessed on the Agilent Bioanalyzer 2100 system. Then, paired-end sequencing of the mRNA library was performed on the HiSeq XTen sequencers (Illumina, San Diego, CA, United States). Single-end sequencing of the miRNA library was conducted on the Illumina HiSeq 2500 (LC-BIO, Hangzhou, China).

The quality control of the original sequencing data (raw reads) was evaluated by FastQC V0.11.5, and then Cutadapt V1.14 was used to remove adaptors (cutoff value Q lower than 20). The clean reads were obtained using Trimmomatic V0.36 after removing low-quality bases at both ends. Thus, a mean of 57,348,784 reads with a Q30 ratio higher than 96.41% for mRNA was obtained. A standard of 25708013 reads with a Q30 ratio greater than 95.94% for miRNA was achieved (Supplementary Tables 1, 2). Next, the clean reads were compared with the reference genome through miRbase for miRNA and HISAT2 V2.1.0 for mRNA. Hence, the unique value of mapping for mRNA is listed in Supplementary Table 3. Additionally, the gene expression was expressed as counts for miRNA or transcripts per million (TPM) for mRNA. TPM is the normalized count of mRNA, which considers sequencing depth and gene length simultaneously.

Our RNA sequencing data were submitted to Gene Expression Omnibus (GEO), with the accession number GSE199508.

### mRNA and microRNAs profiling

The differential expression of mRNA and miRNA in the rats with 2VO and sham rats was separately evaluated by the DESeq2 V1.12.4 and by the edgeR V3.18.1. The read counts were normalized to TPM for mRNA and to reads per million (RPM) for miRNA before the analysis. A threshold was set for filtering out low-expressed miRNAs with the RPM value less than 0.05 and TPM less than 1. The DEMs and mRNAs (DEGs) were statistically analyzed by setting a *P*-value as 0.05 and the Log_2_ (foldchange) absolute value of more than 1. Then, the results of the DEGs and DEMs were visualized as volcano diagrams and heatmap with hierarchical clustering using the pheatmap program. In addition, the sequence homology of the known DEMs with significant dysregulation was evaluated using the miRBase^1^ database supported by RNACentral.

### Identification of differentially expressed transcription factors and regulatory pairs among differentially expressed microRNAs, differentially expressed transcription factors, and differentially expressed genes

The transcription factors of *Rattus norvegicus* were collected from the AnimalTFDB3.0 database^2^ to identify DETFs from the DEGs of the present transcriptome. The intersection of DEGs and transcription factors from AnimalTFDB3.0 was obtained, followed by a visualization *via* Bioinformatics^3^, an online data processing platform.

As regards the DEMs, target prediction of DEMs was first performed with the help of three databases: miRanda^4^, miRWalk2.0^5^, and TargetScanHumanV7.2^6^. The miRNA-gene regulatory pairs were subsequently generated through the overlap of those predictions, targets, and DEGs. Based on the miRNA-gene regulatory pairs, miRNA-TF regulatory pairs were developed through the extraction of the DETFs from the DEGs regulated by DEMs. As regards the DETFs, the target prediction of TFs was performed using CistromeDB^7^ and hTFtarget^8^, developed based on the data of the ChIP-seq experiments (chromatin immunoprecipitation followed by sequencing), and targets with scores less than 4.0 were excluded. Likewise, the targets of DETFs were overlapped with those of DEGs, generating the TF-gene regulatory pairs. Furthermore, backward prediction based on DEMs for corresponding DETFs was developed to acquire the TF-miRNA regulatory pairs. Briefly, sequences covering the upstream of the transcription start sites (TSSs) for 5 kb and downstream for 1 kb were considered as the putative promoter region of DEMs and then were downloaded from NCBI^9^. Subsequently, those sequences were uploaded into PROMO V3.0.2^10^ for the target-TF prediction of DEMs. Finally, four types of regulatory pairs among DEMs, DETFs, and DEGs were obtained, including miRNA-gene, miRNA-TF, TF-gene, and TF-miRNA regulatory pairs. The acquisition of the regulatory pairs was displayed as a Venn diagram using the online platform Bioinformatics.

### Gene ontology and Kyoto Encyclopedia of Genes and Genomes pathway analyses

The miRNA/TF-associated genes were the focus of gene ontology (GO) and Kyoto Encyclopedia of Genes and Genomes (KEGG) enrichment analyses to discover genes highly associated with VaD and deeply involved in the miRNA/TF regulated network. In detail, miRNA/TF-associated genes were the DEGs jointly regulated by DEMs and DETFs, generated from the three sets, including sets of DEMs prediction targets, sets of DETFs prediction targets, and sets of DEGs. Then, Metascape online platform^11^ was performed to enrich the molecular function, cellular component, biological process, and the KEGG pathways of miRNA/TF-associated genes in *Rattus norvegicus* with a *P*-value less than 0.01 as the cutoff value. The top 20 GO terms and top 10 signaling pathways by KEGG analysis are shown as bar charts and Chordal graph, respectively.

### Construction of the microRNA-transcription factor-gene and protein-protein interaction network

An miRNA-TF-gene (M-T-G) network was constructed based on the aforementioned regulatory pairs among DEMs, DETFs, and miRNA/TF-associated genes, followed by a visualization using Cytoscape V3.8.2^12^ to investigate the gene regulation network associated with miRNAs and TFs in VaD. Since the key nodes exert critical effects on the network, key miRNAs, TFs, and genes were obtained for further analysis. First, key miRNAs and TFs were analyzed by two plug-ins of Cytoscape and scored with several parameters, including closeness centrality (CC), betweenness centrality (BC), average shortest path length, outdegree, and neighborhood connectivity retrieved from CytoHubba, as well as the occurrence provided by Motif-Discover. Second, key genes were selected from the miRNA/TF-associated gene set by integrating the protein-protein interaction (PPI) and CytoHubba analytic methods. The STRING V11.5 software^13^ was used to describe the interactive network within miRNA/TF-associated genes through the setting of the confidence score as 0.7. Then, the PPI network of miRNA/TF-associated genes was assessed by the CytoHubba plug-in. Key genes were selected using several topological parameters, including maximum neighborhood component (MNC), maximal clique centrality (MCC), degree, betweenness, and closeness. Additionally, regulatory loops among key miRNAs, TFs, and genes were displayed after visualization in Cytoscape V3.8.2 (see text footnote 12).

### Quantitative real-time polymerase chain reaction analysis

Total RNA was extracted from the cortex of rats and HEK293 cells using the Ultrapure RNA Kit (CWBIO, Beijing, China). miRNA 1st Strand cDNA Synthesis Kit and miRNA Universal SYBR qPCR Master Mix (Vazyme, Nanjing, China) were used to measure the expression of DEMs in the 2VO rat model. Likewise, the DEGs in the rats with 2VO were also measured using Hiscript^®^III RT SuperMix for qPCR and ChamQ Universal SYBR qPCR Master Mix (Vazyme, Nanjing, China). Quantitative real-time polymerase chain reaction (qRT-PCR) analyses for miRNA and mRNA were performed following the manufacturer’s recommendations. The expression of miRNA and mRNA was calculated by the 2^–Δ^ ^Δ^ ^CT^ method (Yi et al., 2021). The primes in this study were synthesized from Sangon Biotech (Shanghai, China) and are listed in Table 1. U6 small RNA was used as the internal reference for miRNA. *Gapdh* and *ACTB* were used as the reference genes for mRNA.

**TABLE 1:** Primers for qRT-PCR and nucleotide sequences for transfection.

Name	Sequence
miR-145-5p RT	5′-GTCGTATCCAGTGCAGGGTCCGAGG
	TATTCGCACTGGATACGACAGGGAT-3′
miR-122-5p-RT	5′-GTCGTATCCAGTGCAGGGTCCGAGG
	TATTCGCACTGGATACGACCAAACA-3′
miR-21-5p-RT	5′-GTCGTATCCAGTGCAGGGTCCGAGG
	TATTCGCACTGGATACGACTCAACA-3′
miR-5132-5p RT	5′-GTCGTATCCAGTGCAGGGTCCGAGGT ATTCGCACTGGATACGACAGCCTG
U6 RT	GTCGTATCCAGTGCAGGGTCCGAGGTA-3′ TTCGCACTGGATACGACAAAATA
miR-145-5p-F	5′-CGGTCCAGTTTTCCCAGGA-3′
miR-145-5p-R	5′-AGTGCAGGGTCCGAGGTATT-3′
miR-122-5p-F	5′-CGCGTGGAGTGTGACAATGG-3′
miR-122-5p-R	5′-AGTGCAGGGTCCGAGGTATT-3′
miR-5132-5p-F	5′-GATATCGTGGGGCGGTGGA-3′
miR-5132-5p-R	5′-CAGTGCAGGGTCCGAGGT-3′
miR-764-3p-F	5′-AACAAGGAGGAGGCCATAGTG-3′
miR-764-3p R	5′-CAGTGCAGGGTCCGAGGT-3′
U6-F	5′-CAAATTCGTGAAGCGTTCCA-3′
U6-R	5′-AGTGCAGGGTCCGAGGTATT-3′
*Serpine1*-F	5′-GCGTCTTCCTCCACAGCCATTC-3′
*Serpine1*-R	5′-TGTCTCTGTTGGATTGTGCCGAAC-3′
*Nedd4l*-F	5′-CGCCGTGCTGTGAAAGATACCC-3′
*Nedd4l*-R	5′-GTGTGACTTTGTGTTGTGGTTTGGG-3′
*Tpm1*-F	5′-GTAGCATCTCTGAACAGACGCATCC-3′
*Tpm1*-R	5′-CAGCCTTCTCAGCCTCCTCCAG-3′
*Pxn*-F	5′-CAGCCAGCAGCAGACCAGAATC-3′
*Pxn*-R	5′-ACTGCCGCTCTACATCCACTCTC-3′
*Plec*-F	5′-CACCGCACTGAACTCGCTACAC-3′
*Plec*-R	5′-GCTCGGCATCTTGGTCACTCTG-3′
*Trip12*-F	5′-CCGAATCAACTGGTGCCGAAGAG-3′
*Trip12*-R	5′-AGGAGGAAGTAGAGGCAGCAGAAG-3′
*Col1a1*-F	5′-TGTTGGTCCTGCTGGCAAGAATG-3′
*Col1a1*-R	5′-GTCACCTTGTTCGCCTGTCTCAC-3′
*Klf5*-F	5′-AGACGAGACAGTGCCTCAGTGG-3′
*Klf5*-R	5′-GCCAGTTCTCAGGTGCGTGATG-3′
*Csrnp1*-F	5′-ACGCTTCTGTAGAGGAGGACTTGG-3′
*Csrnp1*-R	5′-CCGAGGTGGATAGGGCTGTAGG-3′
*Rxrg*-F	5′-CGAATCCTACGGCGACATGAGTG-3′
*Rxrg*-R	5′-AACAAGGGTGAAGAGCTGCTTATCC-3′
*Klf4*-F-F	5′-GGACGGCTGTGGGTGGAAATTC-3′
*Klf4*-R	5′-TGTCGCACTTCTGGCACTGAAAG-3′
*Nfatc4*-F	5′-GCGGTCGTGTTCTTGAGTGTCC-3′
*Nfatc4*-R	5′-CAAAGCCTTCTGGTGGAGGGTAATC-3′
*Foxj2*-F	5′-ACCCGCAAGCCTCTCATCTCTAC-3′
*Foxj2*-R	5′-GTGGTGTGTTGGGCGACTGTATC-3′
*Nkx6-1*-F	5′-AGAGTCAGGTCAAGGTCTGGTTCC-3′
*Nkx6-1*-R	5′-ATCGTCGTCGTCCTCCTCGTTC-3′
*Prdm6*-F	5′-CCACCACCCTCAACAACCACATC-3′
*Prdm6*-R	5′-GTCTTCCAAACGCTTCAGCAAACAG-3′
*Mxi1*-F	5′-GCGAGAGGAGATTGAAGTGGATGTG-3′
*Mxi1*-R	5′-TGATGCTGGTGGTACTGATGTTGTC-3′
*Zfp523*-F	5′-CAATGGCAAAGGGCAGCAAGTTG-3′
*Zfp523*-R	5′-AGTGATGGGCAGTGGTGTAGAGG-3′
*Gapdh*-F	5′-CAAGGTCATCCATGACAACTTTG-3′
*Gapdh*-R	5′-GGGCCATCCACAGTCTCCT-3′
*KLF5-F*	5′-AGTGCCTCAGTCGTAGACCAGTTC-3′
*KLF5-R*	5′-GCCAGTTCTCAGGTGAGTGATGTC-3′
*CSRNP1-F*	5′-ATGCCATTGATGACGCCTCTGTG-3′
*CSRNP1-R*	5′-GGCTGGGTAGGGCTGTAGGAAG-3′
*ACTB-R*	5′-ATACTCCTGCTTGCTGATCC-3′ ATACTCCTGCTTGCTGATCC ATACTCCTGCTTGCTGATCC
*ACTB-F*	5′-CCTGTACGCCAACACAGTGC-3′ CCTGTACGCCAACACAGTGC CCTGTACGCCAACACAGTGC
miR-145-5p mimics	sense 5′-GUCCAGUUUUCCCAGGAAUCCCU-3′
	antisense 5′-GGAUUCCUGGGAAAACUGGACUU-3′
miR-122-5p mimics	sense 5′-UGGAGUGUGACAAUGGUGUUUG-3′
	antisense 5′-AACACCAUUGUCACACUCCAUU-3′
Negative control (NC)	sense 5′-UUCUCCGAACGUGUCACGUTT-3′
	antisense 5′-ACGUGACACGUUCGGAGAATT-3′

### Cell culture and transfection

Human embryonic kidney (HEK293) cells were cultured in DMEM supplemented with 10% FBS (Gibco/Invitrogen, Grand Island, NY, United States) and were incubated following the instructions of the American Tissue Culture Collection. HEK293 cells were seeded into 24-well plates and 6-well plates for transfection after reaching 70% growth confluence using Lipofectamine 2000 (Invitrogen, Carlsbad, CA, United States). Mimics were purchased from Genepharma (Suzhou, China) and were transfected into HEK293 cells to evaluate the regulatory relationship of miR-145-5p over *Csrnp1* and miR-122-5p over *Klf5*; the sequences of mimics and negative control (NC) are listed in Table 1.

### Dual-luciferase reporter assay

Dual-luciferase reporter experiment was used to verify the direct action between miR-145-5p and miR-122-5p over *Csrnp1* and *Klf5*. The binding sequences in the 3′UTR of *Csrnp1* and *Klf5* were provided by miRWalk (see text footnote 5) and cloned into the pmirGLO plasmid vector (Sangon Biotech, Shanghai, China) after amplification. Subsequently, plasmids containing wild-type (WT) binding sequences were transfected into HEK293 cells along with miR-145-5p mimics or miR-122-5p mimics, using NC as the control group. The plasmids with the mutant-type (MUT) binding sequences were also synthesized and transfected into HEK293 cells along with miR-145-5p mimics, miR-122-5p mimics, or NC. The cells were collected at 48 h after transfection and analyzed using the Dual-Luciferase Reporter Assay Kit (Vanzyme, Nanjing, China) under the manufacturer’s recommendations. The activity of renilla luciferase was considered as the endogenous control of firefly luciferase.

### Dataset acquisition and data processing

Gene expression datasets of miRNA and mRNA related to VaD were selected from the GEO^14^, with the species limitation (human, mouse, and rat) and the biological sample type (blood and different brain regions). Ten GEO datasets containing the expression level of miRNA associated with VaD were selected, including GSE193012, GSE178500, GSE86291, GSE100488, GSE111794, GSE48028, GSE184975, GSE29287, GSE46266, and GSE46269. Meanwhile, 22 GEO datasets of mRNA expression status involved in VaD were collected, including GSE104381, GSE201482, GSE186798, GSE80681, GSE60820, GSE97537, GSE202659, GSE173544, GSE173714, GSE131193, GSE45703, GSE163614, GSE106680, GSE21136, GSE157628, GSE111782, GSE134257, GSE107983, GSE37777, GSE17929, GSE162072, and GSE148841. The significantly dysregulated genes were obtained by setting the absolute value of Log_2_ (foldchange) greater than 1 and the *P*-value less than 0.05.

### Statistical analysis

IBM SPSS Statistics (version 25.0, IBM, NY, United States) was used for analyzing the escape latency and the swimming speed in the MWM test with ANOVA, followed by Tukey’s *post hoc* analyses among groups. The unpaired *t*-test was used for the remaining statistical analyses using the GraphPad Prism version 8.0 software (GraphPad Inc., CA, United States). Results are expressed as mean ± standard deviation (SD). Besides, unpaired *t*-test and fold change analyses were applied to distinguish DEMs and DEGs among rats with 2VO and sham rats with a threshold set as *P* < 0.05 and Log_2_ (foldchange) absolute value greater than 1.

## Results

### Spatial cognition and neuronal survival are declined in rats with vascular dementia

Rats with 2VO showed a longer escape latency in the MWM test than the sham rats (Figure 2B, *P* < 0.001), while the swimming speed was not different between these two groups (Figure 2C), indicating that the decline in the learning ability in rats with 2VO was not due to the influence of motor ability. Moreover, rats with 2VO showed less crossings of the target platform (Figure 2D, *P* < 0.001) and shorter duration in the target quadrant than sham rats (Figure 2E, *P* < 0.01), suggesting the occurrence of the loss of memory in rats with 2VO. Furthermore, fewer Nissl bodies were found in the cortex of rats with 2VO (Figures 2F,G, *P* < 0.05 *vs.* sham rats). Together, these results supported the cognitive deficits and neuronal damage in the VaD model.

### Identification of differentially expressed microRNAs and differentially expressed genes related to vascular dementia

**FIGURE 3:**
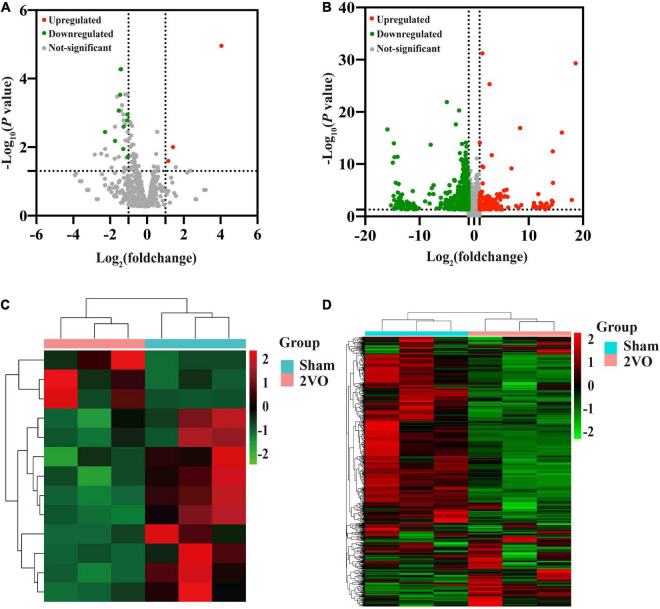
Volcano plots of the transcriptome and hierarchical cluster analysis of the DEMs and DEGs in the cortex of 2VO and sham rats. **(A)** Volcano plots of the differentially expressed miRNAs (DEMs) in the cortex of rats with 2VO compared with sham rats. **(B)** Differentially expressed mRNAs (DEGs) in the cortex of rats with 2VO compared with sham rats. Red dots indicate the upregulated DEMs and DEGs in the brains of rats with 2VO, while green dots indicate downregulated DEMs and DEGs. The molecules with no statistically different changes in rats with 2VO are shown as gray dots. **(C)** Hierarchical clustering analysis of DEMs. **(D)** Hierarchical clustering analysis of DEGs. The *X*-axis shows the cluster analysis of samples, the top blue bar indicates the sham group, and the top red bar indicates the group of rats with 2VO. The *Y*-axis shows the cluster analysis of miRNAs or mRNAs. Upregulated DEMs and DEGs are shown in red, and downregulated DEMs and DEGs are shown in green.

Thirteen known DEMs and 805 DEGs were identified between rats with 2VO and sham rats, including ten downregulated and three upregulated DEMs and 602 downregulated and 203 upregulated DEGs. The volcano diagram illustrated the distribution of DEMs and DEGs acquired from the cortex of the rats with 2VO compared to the sham ones (Figures 3A,B). Further hieratical clustering analysis revealed a differential sequencing expression profiling of these DEMs and DEGs among samples, as shown in Figures 3C,D. Moreover, among the DEMs, ten miRNAs were conserved among humans, mice, and rats according to miRBase (see text footnote 1), while miR-5132-5p, miR-764-3p, and miR-802-5p were not conserved (Table 2).

**TABLE 2:** Sequence and sequence homology of DEMs.

Name	Mature sequence	Log_2_ (foldchange)	*P*-value	Sequence homology
miR-21-5p	5′-UAGCUUAUCAGACUGAUGUUGA-3′	–2.2700	0.017800	rat, mouse, human
miR-511-3p	5′-AAUGUGUAGCAAAAGACAGGA-3′	–1.7300	0.003990	rat, mouse, human
miR-145-5p	5′-GUCCAGUUUUCCCAGGAAUCCCU-3′	–1.5300	0.030800	rat, mouse, human
miR-10b-5p	5′-CCCUGUAGAACCGAAUUUGUGU-3′	–1.4600	0.056800	rat, mouse, human
miR-143-3p	5′-UGAGAUGAAGCACUGUAGCUCA-3′	–1.4100	0.001360	rat, mouse, human
miR-214-3p	5′-ACAGCAGGCACAGACAGGCAG-3′	–1.2800	0.001750	rat, mouse, human
miR-764-3p	5′-GAGGAGGCCAUAGUGGCAACUGU-3′	–1.2700	0.002600	rat, mouse
miR-10a-5p	5′-UACCCUGUAGAUCCGAAUUUGUG-3′	–1.0600	0.000464	rat, mouse, human
miR-223-3p	5′-UGUCAGUUUGUCAAAUACCCC-3′	–1.0500	0.010400	rat, mouse, human
miR-155-5p	5′-UUAAUGCUAAUUGUGAUAGGGGU-3′	–1.0400	0.005740	rat, mouse, human
miR-122-5p	5′-UGGAGUGUGACAAUGGUGUUUG-3′	4.0500	0.011800	rat, mouse, human
miR-5132-5p	5′-CGUGGGGCGGUGGACCCAGGCU-3′	1.4200	0.015700	rat, mouse
miR-802-5p	5′-UCAGUAACAAAGAUUCAUCCU-3′	1.1500	0.039800	rat, mouse

### Identification of transcription factors and microRNA-transcription factor regulatory pairs associated with vascular dementia

**FIGURE 4:**
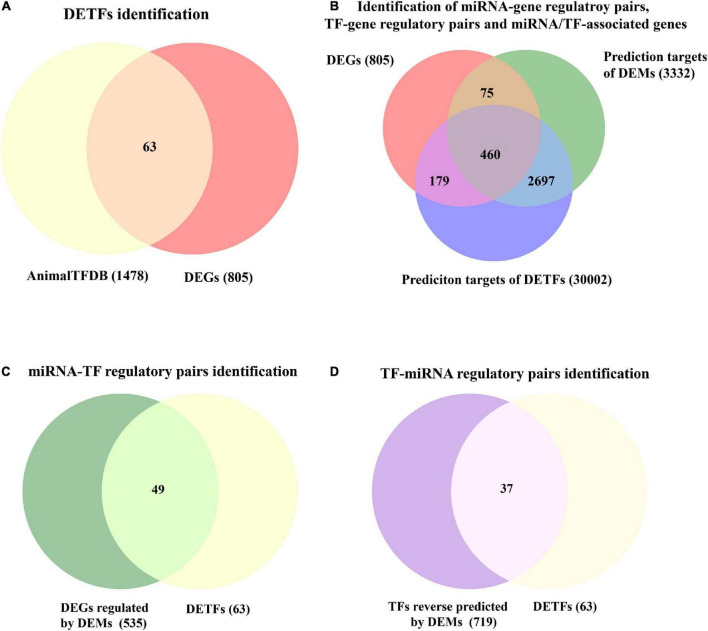
Identification of DETFs and DEGs jointly regulated by DEMs and DETFs. **(A)** Identification of 63 DETFs from DEGs using the Venn diagram. **(B)** Target prediction of DEMs and DETFs. A total of 535 miRNA-gene regulatory pairs, 639 TF-gene regulatory pairs, and 460 miRNA/TF-associated DEGs are identified. **(C)** Identification of 49 miRNA-TF regulatory pairs in total. **(D)** Identification of 37 TF-miRNA regulatory pairs.

A total of 63 VaD-associated DETFs, divided into 18 upregulated and 45 downregulated TFs, were identified among the intersection of 805 DEGs in the cortex of rats with 2VO from RNA sequencing data and 1478 TFs of *Rattus norvegicus* from the AnimalTFDB database, which were useful to investigate the abnormally changed TFs involved in VaD (Figure 4A). The regulatory interactions, the expression fold change, and the *P*-value of 63 disease-related DETFs are listed in Supplementary Table 4.

A total of 3332 putative target genes of 13 DEMs and 30,002 presumptive target genes of 63 DETFs were obtained to investigate the involvement of miRNAs and TFs in the regulation of disease-related genes through a combination of the predicted results from multiple sources, including miRanda, miRWalk2.0, and TargetScanHumanV7.2 for DEMs, and CistromeDB and hTFtarget for DETFs, as described in paragraph 3.7 of the Materials and Methods (Figure 4B). Finally, a total of 460 common miRNA/TF-associated target genes were generated from the intersection among three defined sets of all predicted genes from DEMs, DETFs, and DEGs (Figure 4B).

In addition, the regulatory relationship between miRNAs and TFs related to VaD was also identified. As for miRNA-TF regulatory pairs (the action of miRNA on TF), thirteen DEMs targeted 49 TFs by overlapping 3332 targets of DEMs and 63 DETFs (Figure 4C). Regarding the TF-miRNA regulatory pairs (the action of TF on miRNA), 37 DETFs were identified as having the regulatory effect on 13 DEMs after overlapping 63 DETFs and 719 TFs through reverse prediction of DEMs using the PROMO database (Figure 4D).

### Gene ontology and pathway enrichment analyses

**FIGURE 5:**
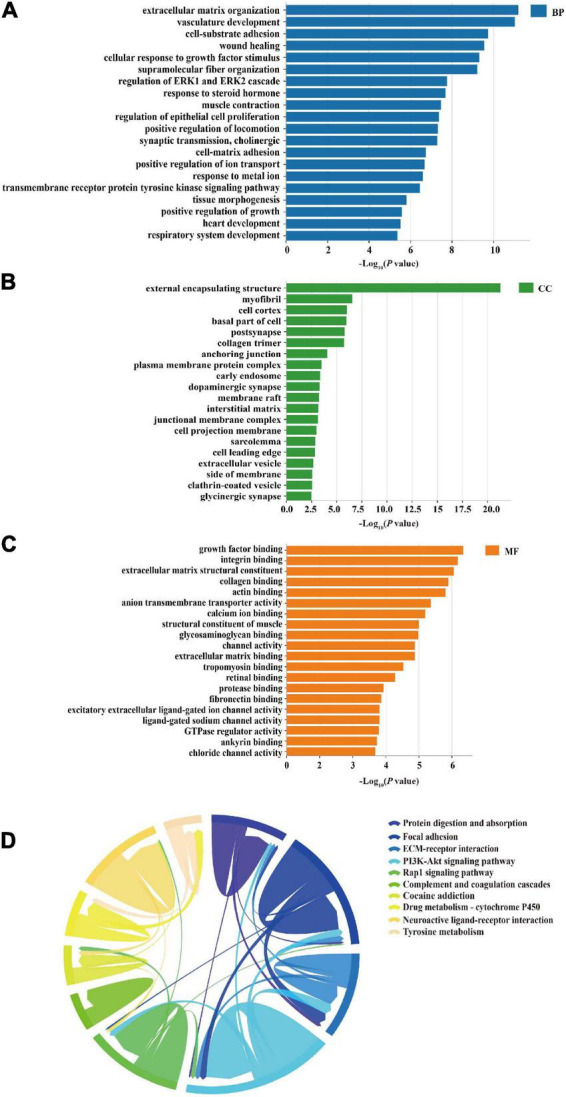
Gene ontology (GO) and KEGG enrichment analyses. **(A)** Top 20 GO terms of biological process (BP) as enrichment of 460 miRNA/TF-associated genes, including vasculature development and regulation of ERK1 and ERK2 cascade. **(B)** Top 20 GO terms of cellular components (CC) from the enrichment analysis of the 460 genes, including myofibril and cell cortex. **(C)** Bar charts of molecular function (MF) enrichment of the 460 genes, including growth factor binding and integrin binding. **(D)** Top 10 signaling pathways of KEGG enrichment analysis presented in the form of a Chordal graph, such as the PI3K-Akt signaling pathway and Wnt signaling pathway. Pathways are distinguished by different colors. The top 10 pathways are listed on the right side according to the *P*-value (from top to bottom in ascending order). Each line represents the association between two pathways. The thicker the line, the more common enriched genes between the two pathways and the stronger the association.

The enrichment analysis of 460 target genes, including aspects of biological process (BP), cell composition (CC), and molecular function (MF), was performed to evaluate the biological function of these common miRNA/TF-associated target genes. Vasculature development, cell-substrate adhesion, wound healing, cellular response to growth factor stimulus, regulation of ERK1, ERK2 cascade, and cholinergic synaptic transmission were included in the top 20 items of BP category, as shown in Figure 5A. The most enriched results of CC were oriented to the cell membrane and cell organelles, such as myofibril, cell cortex, plasma membrane protein complex, early endosome, and dopaminergic synapse (Figure 5B). The main enrichment processes of MF were related to growth factor binding, integrin binding, extracellular matrix structural constituent, collagen binding, actin binding, anion transmembrane transporter activity, and calcium ion binding (Figure 5C).

A total of 37 KEGG pathways were discovered, and the top 10 pathways were obtained after removing uncorrelated pathways such as “cancer” according to a *P*-value < 0.01. The results indicated that these common targets were mostly closely related to the PI3K-Akt signaling pathway, neuroactive ligand-receptor interaction, calcium signaling pathway, and Wnt signaling pathway, which were mainly associated with apoptosis, hypoxia, inflammation, angiogenesis, and neurodegeneration involved in VaD (Figure 5D).

### Construction of the microRNA-transcription factor-gene regulatory network and protein-protein interaction network

**FIGURE 6:**
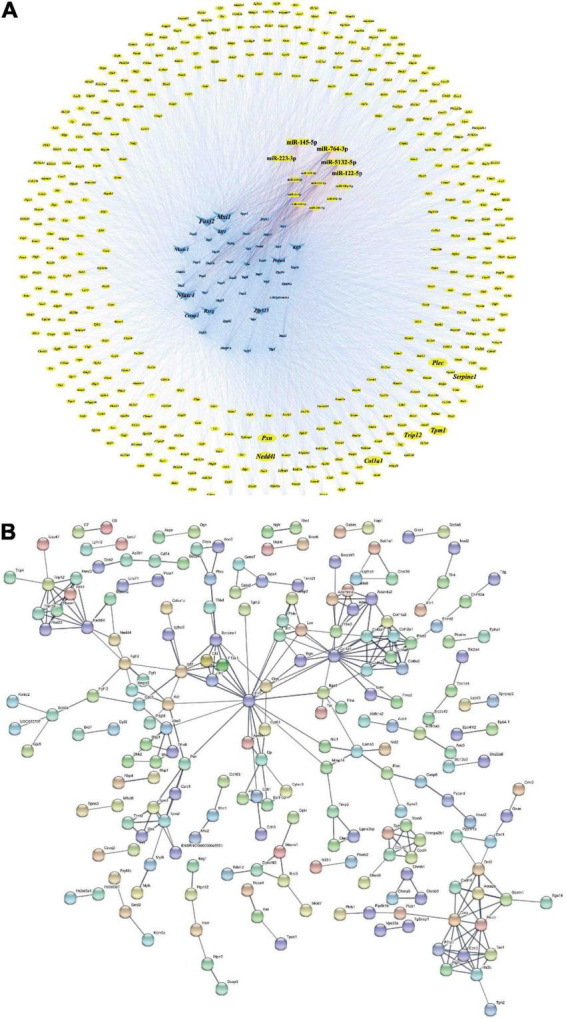
M-T-G and PPI networks. **(A)** Establishment of the M-T-G network of 13 DEMs, 49 DETFs, and 460 DEGs. The genes, miRNAs, and TFs are represented by yellow circles, red rectangles, and blue triangles, respectively. The key nodes are highlighted with larger sizes. **(B)** Establishment of the PPI network based on the 460 joint targets of DEMs and DETFs.

Transcription factors and genes associated with VaD, 13 DEMs, 460 miRNA/TF-associated target genes, and 49 VaD-associated DETFs were involved in the M-T-G network to elucidate the regulatory relationship among these miRNAs. The other 14 VaD-associated TFs were excluded from the network due to their poor association with these acquired miRNA/TF-associated target genes. Finally, the M-T-G network was constructed, resulting composed of 522 nodes, 7537 edges, and four types of regulatory relationships, including the miRNA-gene, TF-gene, miRNA-TF, and TF-miRNA actions (Figure 6A). Additionally, edges projected from DEMs were colored red, while DETFs were blue. Next, 460 miRNA/TF-associated target genes were uploaded into the STRTING software, and the interactions scored under 0.7 were removed, generating a functional protein association network with 205 nodes and 304 edges (Figure 6B). Furthermore, dysregulated key genes were identified by integrating the results from STRING and cytohubba plug-in, including *Serpine1*, *Pxn*, *Nedd4l*, *Col1a1*, *Plec*, *Trip12*, and *Tpm1* (Table 3).

**TABLE 3:** Summary of key genes.

Name	Type	Log_2_ (foldchange)	*P*-value	*Z*-score
*Plec*	gene	–3.5300	0.006660	0.775000
*Col1a1*	gene	–2.0100	0.000001	1.680000
*Pxn*	gene	–1.9300	0.001040	1.280000
*Serpine1*	gene	–1.4300	0.034000	0.732000
*Tpm1*	gene	4.8400	0.041300	0.756000
*Nedd4l*	gene	3.8700	0.002240	0.940000
*Trip12*	gene	2.5500	0.000124	0.578000

### Identification of the involved feed-forward loop and key nodes

miRNAs and TFs are important regulatory elements for gene expression. One of the most common regulatory patterns among miRNAs, TFs, and genes is the three-node regulatory motif composed of the feed-forward loop (FFL) and composite loop (Lin et al., 2015). Specifically, miRNA and TF simultaneously regulate one target with a single direction action within the three-node FFL motif, while their regulation on the common target is bilateral in the three-node composite motif (Zhang et al., 2015).

**FIGURE 7:**
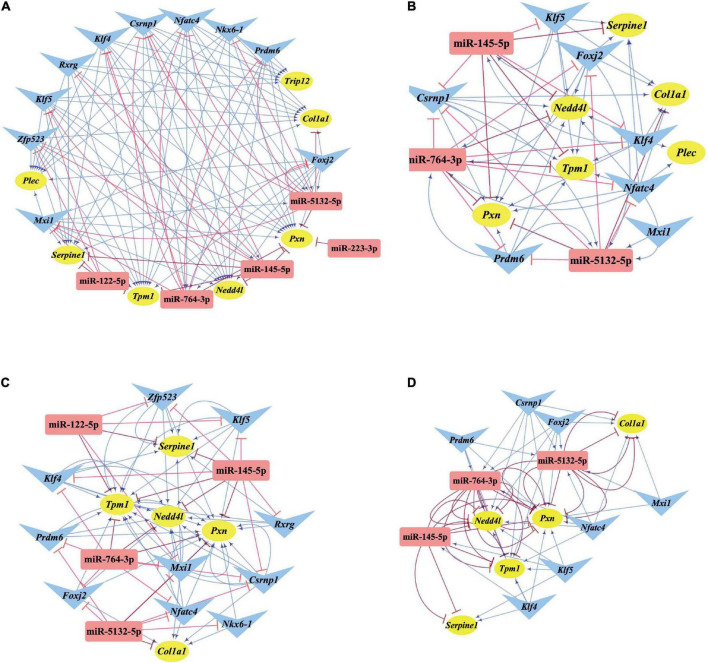
Regulatory relationships of the M-T-G subgraph network, composite loops, and FFLs. **(A)** Establishment of the M-T-G subgraph network of the key nodes based on 7 DEGs, 10 DETFs, and 5 DEMs. **(B)** Summary of all the composite loops based on the M-T-G subgraph network. **(C)** Collection of the miRNA-mediated FFLs extracted from the M-T-G subgraph network. **(D)** Gathering of TF-mediated FFLs of key DETFs. The target genes, miRNAs, and TFs are represented with yellow circles, red rectangles, and blue triangles, respectively. The edges radiated from miRNAs are marked in red and T-shaped blunt arrow ends, while the edges projecting from TFs are marked in blue and sharp arrow ends.

**FIGURE 8:**
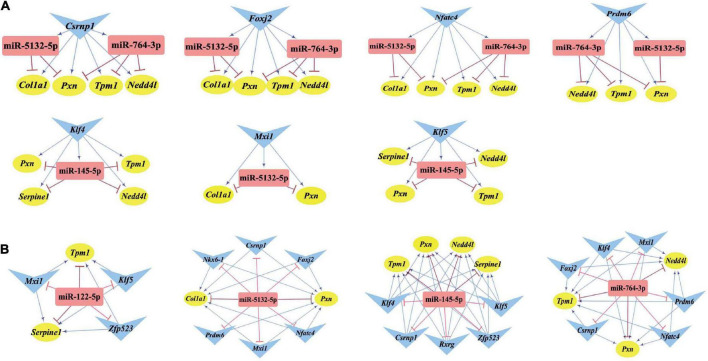
Three-node FFLs formed by key nodes. **(A)** TFs-mediated three-node FFLs of individual key DETFs. **(B)** A total of four miRNA-mediated three-node FFLs of key DEMs of the present study. The gene, miRNA, and TF are represented with yellow circles, red rectangles, and blue triangles, respectively. The terminal of the edges radiating from the miRNA is marked in red and T-type blunt arrow, while the one that changed to sharp arrow for the edges that radiate from TF is in blue color.

**FIGURE 9:**
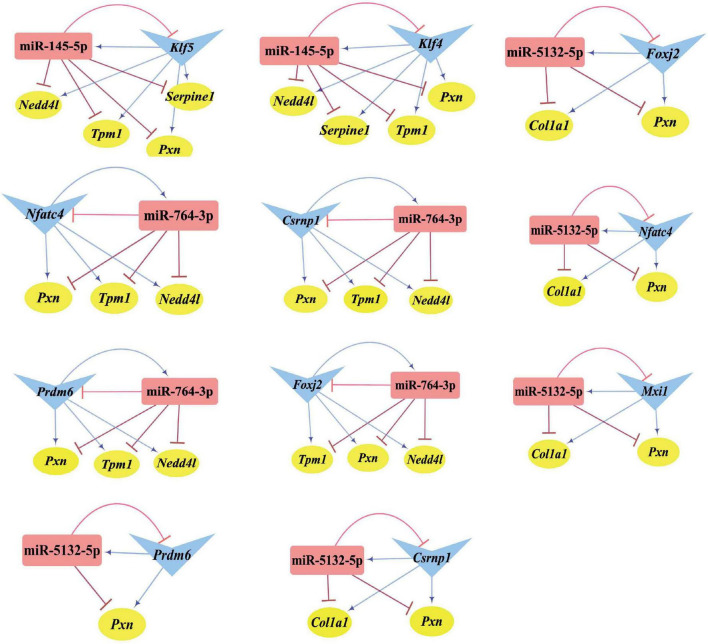
Composite loops among key miRNAs, TFs, and genes. Collection of composite loops of key nodes based on mutual regulatory miRNA-TF pairs. The gene, miRNA, and TF are represented with yellow circles, red rectangles, and blue triangles, respectively. The terminal of the edges radiating from the miRNA is marked in red and T-type blunt arrow, while the one that changed to sharp arrow for the edges radiated from TF is in blue color.

Differentially expressed transcription factors, DEGs, key miRNAs, and TFs were analyzed based on the aforementioned M-T-G network using CytoHubba and Motif-Discovery plugins to further investigate the regulatory motifs of DEMs in VaD. In the present analysis, those key nodes were selected according to the scores measured by the topological properties described in the Materials and Methods and explained in detail in Tables 3, 4. The top 10 of the 63 DETFs were selected, such as *Mxi1*, *Nfatc4*, *Rxrg*, *Zfp523*, *Foxj2*, *Nkx6*-1, *Klf4*, *Klf5*, *Csrnp1*, and *Prdm6*. Top 5 miRNAs of the 13 DEMs were selected, such as miR-5132-5p, miR-764-3p, miR-223-3p, miR-145-5p, and miR-122-5p. Then, an M-T-G subgraph was established based on the key nodes and included 7 DEGs, 10 DETFs, and 5 DEMs (Figure 7A). Subsequently, three-node FFLs were constructed based on the regulatory relationships among these key nodes. The composite loop with key nodes is shown in Figure 7B. Three-node FFLs, including miRNA-dominating and TF-dominating FFLs, are shown in Figures 7C,D. Finally, eleven FFLs mediated by key TFs or key miRNAs and eleven composite loops were constructed (Figures 8, 9). These loops composed of key nodes represented a highly mutual interaction, demonstrating a complex regulatory network in the pathology of VaD.

**TABLE 4:** Summary of key TFs and miRNAs.

Name	Type	Occurrences	*Z*-score
*Mxi1*	transcription factor	47100	1.4600
*Nfatc4*	transcription factor	150000	1.4000
*Rxrg*	transcription factor	162000	1.3700
*Zfp523*	transcription factor	64700	1.3600
*Foxj2*	transcription factor	28700	1.3600
*Nkx6-1*	transcription factor	35200	0.7900
*Klf4*	transcription factor	51000	0.6140
*Klf5*	transcription factor	104000	0.4840
*Csrnp1*	transcription factor	53600	0.3760
*Prdm6*	transcription factor	39300	0.3620
miR-5132-5p	miRNA	2.2200	1.4200
miR-764-3p	miRNA	1.6300	1.0700
miR-223-3p	miRNA	0.9200	0.9090
miR-145-5p	miRNA	0.5090	0.8110
miR-122-5p	miRNA	0.7340	0.5950

### Verification of key nodes and regulatory relationship

Before the experimental verification of the expression or interaction among key nodes in the M-T-G network, the expression pattern of these key nodes (key miRNAs, key TFs, and key genes) was assessed using several published GEO datasets associated with VaD from different types of biological samples of several organisms such as human, rat, and mouse. Supplementary Table 5 shows that four key miRNAs, such as miR-145-5p, miR-122-5p, miR-223-3p, and miR-764-3p, were dysregulated in several brain regions of rodent models related to VaD, including the hippocampus, cerebral cortex, striatum, and subventricular zone. Among them, miR-145-5p, miR-122-5p, and miR-223-3p in the hippocampus of mice suffering from VaD-related disease displayed similar expression patterns to our sequencing analysis. Additionally, two key TFs, *Klf4* and *Foxj2*, were significantly downregulated in the hippocampus of the mouse models of cerebral ischemia, as consistent with our analysis. Moreover, five key TFs, such as *Klf5*, *Csrnp1*, *Mxi1*, *Rxrg*, and *Zfp523*, were significantly dysregulated in the blood or cortical tissues of rodent models related to VaD, as shown in Supplementary Table 6. *Serpine1* was significantly downregulated in the hippocampus under the VaD-related pathological conditions. The other five key genes, *Tpm1*, *Col1a1*, *Pxn*, *Nedd4l*, and *Plec*, were significantly dysregulated in the rodent model related to VaD, aligned with our RNA sequencing analysis (Supplementary Table 7).

**FIGURE 10:**
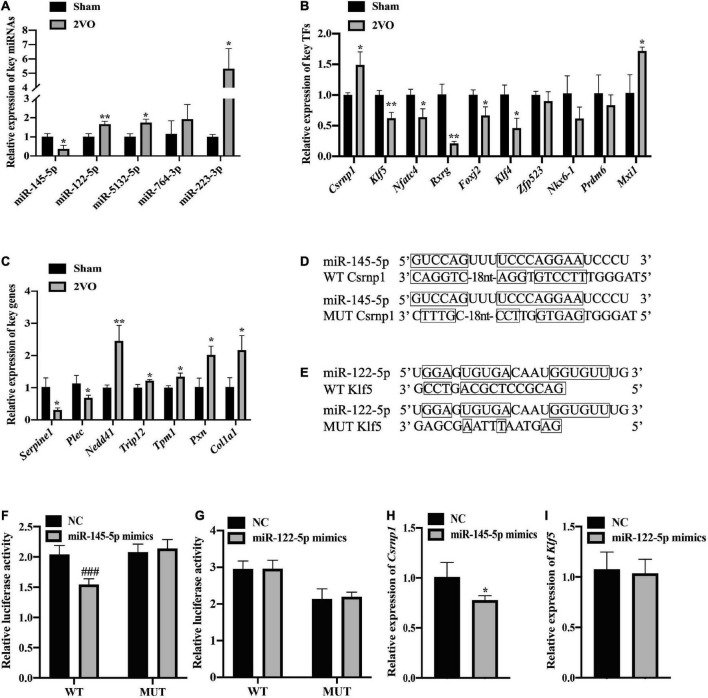
Validation of the expression and regulatory relationship. **(A)** Verification of the differential expression of the key miRNAs using qRT-PCR (*n* = 3). **(B)** Relative expression of the key TFs in the cortex of the rats with 2VO compared with the sham rats using qRT-PCR analysis (*n* = 3). **(C)** Relative expression of the key genes using qRT-PCR analysis (*n* = 3). **(D)** Wild-type (WT) or mutant-type (MUT) binding sites for the combination of miR-145-5p and *Csrnp1* in the dual-luciferase reporter assay. **(E)** Binding sites of the wild or mutant type of miR-122-5p and *Klf5*. **(F)** Direct interaction of miR-145-5p/*Csrnp1* by dual-luciferase reporter assay (*n* = 6). **(G)** No direct interaction of miR-122-5p/*Klf5* by dual-luciferase reporter assay (*n* = 6). **(H)** Significant decrease of the *Csrnp1* expression in the presence of miR-145-5p mimics. **(I)** No difference in the *Klf5* expression in the presence of miR-122-5p. Results are presented as mean ± SD. *P < 0.05, **P < 0.01 *vs*. Sham. ^###^*P* < 0.001 *vs*. NC.

Quantitative real-time polymerase chain reaction analysis of the genes in the cortical tissues of rats with 2VO and sham rats was performed to confirm the changes in the expression of these key nodes in the predicted FFLs. In these detected nodes, the differential expression of key miRNAs, including miR-145-5p, miR-122-5p, and miR-5132-5p, TFs including *Csrnp1*, *Klf4*, *Nfatc4*, *Rxrg*, *Foxj2*, and *Klf5*, and target genes including *Serpine1*, *Plec*, *Nedd4l*, *Trip12*, and *Tpm1* between rats with 2VO and sham rats were in line with the results analyzed by RNA sequencing (Figures 10A–C, *P* < 0.05-0.01 *vs.* sham). Although the expression of several key nodes, including *Nkx6*-1, *Prdm6*, and *Zfp523*, were in line with the sequencing results, the statistical difference in rats with 2VO was not significant as compared with sham rats. In addition, some of the predicted key nodes completely deviated from the sequencing results, including miR-764-3p, miR-223-3p, *Mxi1*, *Pxn*, and *Col1a1* (all *P* < 0.05 *vs.* sham). Since the changing trend of most predicted key nodes was similar to that in the sequencing analysis, our speculation was that the abnormal expression of these key miRNAs and genes in VaD within the FFLs was reliable.

As for the validation of the analyzed regulatory relationship, two key DEMs (miR-145-5p and miR-122-5p) were chosen to verify the miRNA-TF regulatory pairs thanks to their excellent sequence conservation, consistency of the expression pattern, and associations with the brain injury caused by hypoxia (Li et al., 2020b; Yang et al., 2021). *Csrnp1* and *Klf5* were then selected, since they are the targets of miR-145-5p and miR-122-5p due to their most significant different expression according to the value of Log_2_ (foldchange). The construction of recombinant plasmids containing MUT binding sites or WT binding sites for luciferase assessment is shown in Figures 10D,E. The dual-luciferase reporter assay revealed that miR-145-5p directly targeted *Csrnp1*, which was in line with the bioinformatic analysis of their potential relationship within the FFL (Figures 10F,G, *P* < 0.001 *vs*. NC). However, the connection between miR-122-5p and *Klf5* was not found in the dual-luciferase reporter experiment. Furthermore, the expression of *Csrnp1* in HEK293 cells was significantly downregulated in the presence of miR-145-5p mimics (Figures 10H,I, *P* < 0.05 *vs*. NC), while no statistical difference was found in the expression of *Klf5* when transfected with miR-122-5p mimics compared to the NC. Thus, miR-145-5p-*Csrnp1*, the key miRNA-TF regulatory pair in the predicted FFL involved in VaD, was obtained through qRT-PCR analysis and target verification.

## Discussion

Vascular dementia is a severe progressively cognitive dysfunction troubling a growing population of the elderly and without curative treatment or definite criteria for diagnosis. Thus, an early intervention on the disease and the control of risk factors are crucial steps in reducing the incidence of dementia (Morovic et al., 2019). The pathology of VaD refers to multiple processes, such as endothelial dysfunction, blood-brain barrier (BBB) destruction, and neuroinflammation, suggesting that an approach focusing on a single target is inappropriate (Cipollini et al., 2019). Therefore, a deep understanding of the aberrant genetic changes and their associated molecular mechanisms involved in the VaD pathophysiology may provide new therapeutic methods.

In the present study, rats with 2VO were used as a model of VaD, which exhibited cognitive impairments and neuropathological changes that spread in the brain, such as neuronal apoptosis, synaptic loss, and failure of neuronal signaling (Farkas et al., 2007). There is evidence supporting that the cerebral cortex participates in learning and memory, coupling with the hippocampus to mediate memory consolidation (Maingret et al., 2016; Wais et al., 2018). The cortex may be more vulnerable to BBB leak induced by biochemical mediators of cerebral hypoperfusion than other brain regions (Tayler et al., 2021). Furthermore, the risk of VaD may be higher for men than for women with the increase in age (Ruitenberg et al., 2001). Considering the possible interference of gender on the heterogeneity of results, our study detected the learning and memory-behavioral decline and cortical histopathological changes in the neurons of male rats suffering from 2VO; subsequently, DEMs, DETFs, and DEGs in the etiology of VaD were discovered using RNA sequencing in these rats combined with bioinformatics and experimental analyses to identify novel potential targets and understand their molecular mechanisms in VaD.

Among these differentially expressed genes, 805 DEGs were obtained based on the transcriptome sequencing and included 602 downregulated and 203 upregulated mRNAs. Seven genes were defined as key genes according to the M-T-G network and PPI network for their tight associations with the regulation of miRNAs and TFs in VaD. Among these seven key genes, the involvement of *Col1a1*, *Plec*, and *Trip12* in VaD was reported for the first time. As regards other key genes, *Serpine1* was significantly downregulated in VaD according to the present study and was also downregulated in small vessel brain injury as previously reported, which is a significant vascular factor for cognitive impairment (Knottnerus et al., 2010; Pantoni, 2010). Moreover, *Serpine1* was downregulated not only in the cortical brain of rats with 2VO but also in the hippocampus of the mice under chronic cerebral hypoperfusion based on the analysis of GEO datasets. *Serpine1* exerts neuroprotective effects through the inhibition of the endogenous mitochondrial apoptosis pathway and the stabilization of the neuronal networks depending on the MAPK/ERK pathway (Soeda et al., 2008; Iwaki et al., 2012). Furthermore, *Seprine1* is regulated by miR-301a and HIF-1α in response to hypoxia (Gonsalves et al., 2015). In our study, the expression of novel regulators of *Seprine1* in VaD was found based on sequencing and bioinformatic analysis, such as miR-145-5p, miR-122-5p, *Klf5*, and *Klf4*. Another upregulated key gene, such as *Nedd4l*, was also involved in several FFLs of our study dominated by key miRNAs or TFs, such as miR-145-5p, miR-764-3p, *Csrnp1*, *Klf5*, and *Klf4*. It was also confirmed that *Nedd4l* was significantly upregulated in the brain of rats with acute cerebral ischemia (Kim et al., 2021) and might function in exacerbating the toxicity of accumulated excitatory transmitters out of the cells in ischemia through the regulation of the degradation of glutamate transporters (Sopjani et al., 2010; Vina-Vilaseca and Sorkin, 2010). Moreover, *Nedd4l* was reported as regulated by miR-454 to induce a cardioprotective effect (Wang Y. et al., 2021). Besides, Tpm1 has a potential association with cognitive impairments as it was reported as a potential biomarker of AD for its upregulation in the platelets of AD and MCI patients (Reumiller et al., 2018). Tpm1 was regulated by almost all key miRNAs and TFs in our M-T-G network, indicating that it might play an important role in VaD.

Gene ontology and KEGG pathway analyses were performed to better understand the biological functions and potential mechanisms of the common miRNA/TF-associated targets in the pathogenesis of VaD. GO enrichment analysis indicated that these targets were mainly involved in cell membrane and organelles, including cell cortex, postsynapse, dopaminergic synapse, basal part of cell, plasma membrane protein complex, and early endosome. Functional enrichment analysis revealed important roles of these common targets involved in the biological processes related to synaptic transmission, cell proliferation, migration, and vasculature development. These results partly explained the target functions associated with the pathogenesis of VaD. The KEGG enrichment analysis summarized 37 pathways closely related to VaD, including PI3K-AKT signaling pathway, neuroactive ligand–receptor interaction, calcium signaling pathway, and WNT signaling pathway. It has been reported that the upregulation of the PI3K-AKT signaling pathway exerts a protective function in VaD through the promotion of the expression of BCL-2 (Chen et al., 2018). The activation of the Wnt signaling pathway may improve the cognitive ability of rats with 2VO (Jin et al., 2017). Therefore, these results suggest that these common miRNA/TF-associated targets potentially mediated VaD pathogenesis through multiple signaling pathways. These potential mechanisms of targets in the pathogenesis of VaD require further investigation.

Regarding the miRNAs, thirteen DEMs in the cortices of 2VO rats were identified by the RNA sequencing analyses, indicating their potential function in VaD. Among these DEMs, ten out of thirteen were conserved among different species based on miRBase. Next, five key DEMs were selected from the M-T-G network of VaD, such as miR-122-5p, miR-223-3p, miR-145-5p, miR-5132-5p, and miR-764-3p, which were identified for the first time in VaD. Among these five key DEMs, miR-122-5p, miR-223-3p, and miR-145-5p attracted more attention as they have excellent sequence homology, which has been reported as dysregulated in the blood of patients who have cancer, cardiovascular diseases, and neurodegenerative diseases (Maruyama et al., 2018; Mancuso et al., 2019; Ashirbekov et al., 2020; Pinho et al., 2020; Singh et al., 2020). However, after the experimental verification using qRT-PCR, the expression changes of miR-223-3p and miR-764-3p were inconsistent with the RNA sequencing outcome. Among the key miRNAs with consistent changes with RNA sequencing, miR-5132-5p is reported for the first time as associated with 2VO and upregulated in rats with this disease. Moreover, the level of miR-145-5p and miR-122-5p was dysregulated in the cerebrospinal fluid or plasma of patients with cerebral vascular disease as suggested by the other GEO datasets. miR-145-5p is closely related to various cardiovascular diseases. For example, miR-145-5p suppresses the activation of activated microglia cells in cerebral infarction by targeting the 3′UTR of *PLA2G4A* (Qi et al., 2017). miR-145-5p also protects cardiac microvascular endothelial cells against hypoxia/reoxygenation injury by suppressing *Smad4* expression (Li et al., 2020b). miR-122-5p is involved in the inhibition of cardiac fibroblast differentiation induced by apigenin through targeting the transcription factor HIF-1α (Feng et al., 2021); it is regulated by another transcription factor HNF4α in type 2 diabetic mice (Xu et al., 2020), indicating a regulation between miRNA and TF in different diseases.

As regards the TFs, a total of 63 TFs were differentially expressed in rats with 2VO. Ten DETFs were selected from the M-T-G network as the key TFs, divided into nine downregulated TFs and one upregulated TFs. These ten dysregulated TFs were found in VaD for the first time. *Klf5*, the most downregulated TF, is an active factor associated with miRNAs in cancer and cardiovascular disease (Drosatos et al., 2016; Yang et al., 2018; Nan et al., 2021; Wang Y. et al., 2021). *Klf5* was significantly downregulated in the blood of rats with ischemic stroke obtained from the GSE21136. Additionally, a previous study revealed the negative role of *Klf5* in neuronal apoptosis of ischemic stroke through the JNK pathway (Chang et al., 2020). As the most upregulated key TF, *Csrnp1* is reported as a tumor suppressor factor and mediates cardiomyocyte apoptosis induced by restraint stress through the WNT/β-catenin signaling (Ye et al., 2017). Besides, other RNA-sequencing datasets confirmed the upregulation of *Csrnp1* in VaD in humans, rats, and mice, which was consistent with our analysis. Furthermore, *Csrnp1* is highly associated with hypoxic-ischemic encephalopathy, according to RNA sequencing (Xiong et al., 2020). *Nfatc4* was identified as involved in hippocampal plasticity, axonal growth, neuronal survival, and apoptosis (Bradley et al., 2005). A previous study revealed that *Nfatc4* is essential for BDNF-dependent neuron survival and the spatial memory of hippocamp in adult-born neurons (Quadrato et al., 2012).

As regards the regulatory loops, the M-T-G network was constructed based on 13 DEMs, 49 DETFs, and 460 DEGs. Then, key nodes were identified from the M-T-G network. The regulatory relationships among key nodes were manifested as 11 FFLs and 11 composite loops, illustrating that these identified miRNAs, TFs, and target genes possessed significant predictive values in the involvement in VaD. miR-145-5p and miR-122-5p were significantly dysregulated, as supported by the sequencing, qRT-PCR analysis, and the comprehensive comparison of expression patterns among public datasets associated with VaD. miR-122-5p and miR-145-5p are involved in the pathological process of ischemic stroke through the targeting of *Nurrl* and *SEMA3A*, as previously reported (Xie et al., 2017; Yang et al., 2021). Additionally, miR-145-5p directly regulates the expression of *Samd4* in cardiac microvascular endothelial cell injury induced by hypoxia (Li et al., 2020b). Based on the potential interactions of the constructed FFLs and composite loops, the regulatory relationship of miR-145-5p over *Csrnp1* was further validated by dual-luciferase reporter assay and gene expression using a gain-of-function experiment, in line with the bioinformatic prediction. As the most dysregulated transcription factor, *Csrnp1* was reported to mainly function in tumors or cardiac diseases, but little is known on the role of this gene in VaD. Interestingly, *Klf5* was the most downregulated key transcription factor and targeted by miR-145-5p and miR-122-5p simultaneously, according to miRNA-mediated FFLs. Although the direct interaction of *Klf5* and miR-145-5p was previously established (Liang et al., 2018; Zhou et al., 2019), the correlation of miR-122-5p and *Klf5* was found neither by dual-luciferase reporter assay nor by expression analysis based on miRNA gain-of-function.

Our study is just the beginning of this complex study, since still many challenges and problems should be solved in the future. First, larger sample sizes and more profound mechanistic research are needed to confirm our findings on these identified key miRNAs and TFs. Second, gender factors and more vulnerable brain regions of cerebral hypoperfusion should be the focus of our subsequent work to obtain a comprehensive elucidation of the transcriptome changes for new perspectives to understand the complex pathology of VaD. Third, profiling the transcript features of non-canonical small non-coding RNAs, including rsRNA, tsRNA, and snosRNA, that were obtained in the present sequencing is necessary, referring to the previous report (Shi et al., 2021), since they gained increasing attention as regulators of gene expression and biomarkers of diseases, such as transfer-RNA-derived small RNA (Li et al., 2020c; Zhang et al., 2020; Shi et al., 2022). Finally, more key genes and their interactions should be explored from the constructed FFLs and composite loops with potential functions in the pathogenesis of VaD.

In conclusion, the aberrantly expressed miRNAs, TFs, and target genes related to VaD were identified using RNA sequencing analyses in the cortex of rats with 2VO. The biological functions and potential mechanisms of these identified miRNAs and TFs were analyzed. In line with the bioinformatics analyses, three miRNAs, six TFs, and five mRNAs were confirmed as significantly differentially expressed in rats with VaD, and the interaction of miR-145-5p and *Csrnp1* was verified. Our findings might provide new insights into the molecular mechanism of VaD.

## Data availability statement

Publicly available datasets were analyzed in this study. This data can be found here: the RNA sequencing data used in the present study were submitted to Gene Expression Omnibus and the accession number GSE199508 was obtained.

## Ethics statement

The animal study was reviewed and approved by the Ethics Committee of the Institute of Medicinal Biotechnology, Chinese Academy of Medical Sciences (Beijing, China).

## Author contributions

RL and ZL contributed to the conception and designation of the research, interpreted results, and revised the manuscript. KZ and LZ conducted the miRNA and mRNA profiling analysis and key nodes assays, performed the qRT-PCR and the dual-luciferase reporter experiments, and drafted the manuscript. ZC, ML, and TS performed the M-T-G network analysis and prepared the figures. All authors contributed to manuscript revision, read, and approved the submitted version.

## Footnotes

1
http://www.mirbase.org/

2
http://bioinfo.life.hust.edu.cn/AnimalTFDB/

3
http://www.bioinformatics.com.cn

4
https://www.bioinformatics.com.cn/local_miranda_miRNA_target_prediction_120

5
http://mirwalk.umm.uni-heidelberg.de/

6
http://www.targetscan.org/vert_72/

7
http://www.cistrome.org/db

8
http://bioinfo.life.hust.edu.cn/hTFtarget#!/

9
www.ncbi.nlm.nih.gov/

10
alggen.lsi.upc.es/cgi-bin/promo_v3/promo/promoinit.cgi?dirDB=TF_8.3

11
https://metascape.org/gp/index.html#/main/step1

12
https://www.cytoscape.org/

13
https://www.string-db.org/

14
https://www.ncbi.nlm.nih.gov/gds/
