# Supplementary material for: RNA sequencing-based identification of the regulatory mechanism of microRNAs, transcription factors, and corresponding target genes involved in vascular dementia
Source: Front Neurosci. 2022 Sep 20;16:917489. doi: 10.3389/fnins.2022.917489 (PMC9531238; doi:10.3389/fnins.2022.917489)
Supplement: Supplementary file 1 [file Data_Sheet_1.PDF]

Supplementary Table 1. Quality control data of mRNA sequencing.

Sample	Total Reads (count)	Total Bases Count(bp)	Average Read Length (bp)	Q10 Bases Ratio (%)	Q20 Bases Ratio (%)	Q30 Bases Ratio (%)	N Bases Count (bp)	GC Bases Ratio (%)
sham rat 1	59462754	8607319458	144.75	99.99	99.01	96.45	10622	48.92
sham rat 2	55745440	8071010914	144.78	99.99	99.02	96.46	8679	49.25
sham rat 3	62777070	9097221698	144.91	99.99	99.03	96.52	12232	49.13
rat 1 with 2VO	52520958	7611920518	144.93	99.99	99.01	96.42	8387	49.70
rat 2 with 2VO	55903618	8093061981	144.77	99.99	99.02	96.46	10255	48.88
rat 3 with 2VO	57682866	8329705920	144.41	99.99	98.99	96.41	9914	48.78

Supplementary Table 2. Quality control data of miRNA sequencing.

Sample	Reads (counts)	Unique-reads (counts)	Base (counts)	Average length	Q10 Ratio (%)	Q20 Ratio (%)	Q30 Ratio (%)	GC-percentage (%)
sham rat 1	21987456	756128	478283349	21.75	100	99.11	96.39	49.38
sham rat 2	25217676	944950	549681116	21.8	100	99.12	96.40	48.82
sham rat 3	24623554	733099	536896950	21.8	100	99.19	96.35	48.83
rat 1 with 2VO	25427449	967412	556732209	21.89	100	99.22	96.57	48.58
rat 2 with 2VO	36191743	1083018	789911453	21.83	100	99.06	96.09	48.70
rat 3 with 2VO	20800202	666165	452202594	21.74	100	98.95	95.94	49.07

Supplementary Table 3. Summary of genome mapping for mRNA.

Sample	Total reads	Total mapped	Multiple mapped	Uniquely mapped
sham rat 1	61298712(100.00%)	58573860(95.55%)	4144474(6.76%)	54429386(88.79%)
sham rat 2	54642968(100.00%)	52252261(95.62%)	3827428(7.00%)	48424833(88.62%)
sham rat 3	58130830(100.00%)	55605452(95.66%)	4184838(7.20%)	51420614(88.46%)
rat 1 with 2VO	51145376(100.00%)	49043662(95.89%)	4091675(8.00%)	44951987(87.89%)
rat 2 with 2VO	54798862(100.00%)	52544341(95.89%)	3627705(6.62%)	48916636(89.27%)
rat 3 with 2VO	56607882(100.00%)	54120999(95.61%)	3661793(6.47%)	50459206(89.14%)

Supplementary Table 4. Summary of TFs for the construction of the M-T-G network.

DETFs	Regulation category	Log2(fold change)	*P* value
Arid1b	TF-gene, TF-miRNA, miRNA-TF	17.9648	0.000690
Arntl2	TF-gene, TF-miRNA, miRNA-TF	13.0073	0.024585
Esr1	TF-gene, TF-miRNA, miRNA-TF	11.1674	0.013483
Csrnp1	TF-gene, TF-miRNA, miRNA-TF	6.8721	0.000001
Nfia	TF-gene, TF-miRNA, miRNA-TF	2.8559	0.000001
Meis3	TF-gene, TF-miRNA, miRNA-TF	2.7263	0.000631
Zbtb5	TF-gene, TF-miRNA, miRNA-TF	2.3838	0.025872
Zbtb20	TF-gene, TF-miRNA, miRNA-TF	2.3382	0.000565
Ppard	TF-gene, TF-miRNA, miRNA-TF	2.3114	0.001199
Crebrf	TF-gene, TF-miRNA, miRNA-TF	1.1371	0.002958
Zbtb37	TF-gene, TF-miRNA, miRNA-TF	1.0585	0.044613
Plscr1	TF-gene, TF-miRNA, miRNA-TF	-12.5183	0.049185
Klf5	TF-gene, TF-miRNA, miRNA-TF	-12.3068	0.002431
Ebf1	TF-gene, TF-miRNA, miRNA-TF	-8.1898	0.003383
Rxrg	TF-gene, TF-miRNA, miRNA-TF	-3.5400	0.015200
Ahctf1	TF-gene, TF-miRNA, miRNA-TF	-2.9668	0.020600
Nkx6-1	TF-gene, TF-miRNA, miRNA-TF	-2.9320	0.007081
Prrx2	TF-gene, TF-miRNA, miRNA-TF	-2.8014	0.008397
Prdm6	TF-gene, TF-miRNA, miRNA-TF	-2.1850	0.000227
Alx4	TF-gene, TF-miRNA, miRNA-TF	-2.1592	0.004334
Foxc2	TF-gene, TF-miRNA, miRNA-TF	-1.8904	0.000004
Foxd2	TF-gene, TF-miRNA, miRNA-TF	-1.8594	0.019289
Foxd1	TF-gene, TF-miRNA, miRNA-TF	-1.8385	0.000015
Nr2f2	TF-gene, TF-miRNA, miRNA-TF	-1.7929	0.000080
Tbx18	TF-gene, TF-miRNA, miRNA-TF	-1.7629	0.000001
Bnc2	TF-gene, TF-miRNA, miRNA-TF	-1.7060	0.010178
Dach1	TF-gene, TF-miRNA, miRNA-TF	-1.6769	0.002511
Nfatc4	TF-gene, TF-miRNA, miRNA-TF	-1.3933	0.000145
Rarb	TF-gene, TF-miRNA, miRNA-TF	-1.3632	0.000058
Foxc1	TF-gene, TF-miRNA, miRNA-TF	-1.3626	0.000003
Foxj2	TF-gene, TF-miRNA, miRNA-TF	-1.2920	0.024488
Mxi1	TF-gene, TF-miRNA, miRNA-TF	-1.2277	0.009234
Osr1	TF-gene, TF-miRNA, miRNA-TF	-1.1580	0.000046
Zbtb7a	TF-gene, TF-miRNA, miRNA-TF	-1.1504	0.008153
Six5	TF-gene, TF-miRNA, miRNA-TF	-1.0740	0.000715

Klf4	TF-gene, TF-miRNA，miRNA-TF	-1.0269	0.003038
Tead4	TF-gene, TF-miRNA，miRNA-TF	-1.0093	0.024235
Tal1	TF-gene, miRNA-TF	12.2683	0.002935
Hnf4g	TF-gene, miRNA-TF	10.9896	0.002701
Zfp202	TF-gene, miRNA-TF	5.5217	0.000011
Mkx	TF-gene, miRNA-TF	1.0205	0.003882
Tbx3	TF-gene, miRNA-TF	-13.3627	0.002906
Tfap2a	TF-gene, miRNA-TF	-10.7593	0.012546
Zfp523	TF-gene, miRNA-TF	-5.2327	0.000646
Zfp286a	TF-gene, miRNA-TF	-2.0900	0.011800
LOC103690166	TF-gene, miRNA-TF	-1.8121	0.007012
Zfp384	TF-gene, miRNA-TF	-1.2218	0.018037
Zfp82	TF-gene, miRNA-TF	-1.1528	0.040969
Creb3l1	TF-gene, miRNA-TF	-1.0952	0.026909

Supplementary Table 5. Comparison of key miRNAs with published GEO datasets in VaD-related diseases.

GSE number	Sample organism	Sample type	miRNA name	Log_2(fold change)	*P* value
GSE193012	Homo sapiens	cerebrospinal fluid	miR-145-5p	-4.4098	0.2000
GSE193012	Homo sapiens	cerebrospinal fluid	miR-223-3p	-3.4808	0.1300
GSE178500	Homo sapiens	serum	miR-122-5p	1.4200	0.0400
GSE86291	Homo sapiens	plasma	miR-122-5p	2.4818	0.2453
GSE86291	Homo sapiens	plasma	miR-145-5p	-1.6833	0.1040
GSE100488	Homo sapiens	plasma	miR-223-3p	-1.0500	0.0076
GSE111794	Homo sapiens	carotid artery tissue	miR-145-5p	-1.7930	0.0362
GSE48028	Mus musculus	hippocampus	miR-122-5p	1.1691	0.2841
GSE48028	Mus musculus	hippocampus	miR-145-5p	-0.5760	0.3931
GSE48028	Mus musculus	hippocampus	miR-223-3p	1.4789	0.2626
GSE184975	Rattus norvegicus	striatum	miR-122-5p	2.5304	0.1800
GSE184975	Rattus norvegicus	striatum	miR-764-3p	-3.7384	0.0179
GSE29287	Rattus norvegicus	subventricular zone	miR-145-5p	-1.1300	0.0171
GSE46266	Rattus norvegicus	cortex	miR-223-3p	-2.0691	0.0768
GSE46269	Rattus norvegicus	cortex	miR-223-3p	-1.8875	0.0790

Supplementary Table 6. Comparison of key TFs with published GEO datasets in VaD-related diseases.

GSE number	Sample organism	Sample type	TF name	Log_2(fold change)	*P* value
GSE201482	Homo sapiens	blood	*Csrnp1*	1.2314	0.0005

GSE201482	Homo sapiens	blood	*Nkx6-1*	-1.7440	0.5618
GSE186798	Homo sapiens	astrocyte	*Rxrg*	1.5884	0.0578
GSE80681	Mus musculus	hippocampus	*Nfatc4*	-1.2439	0.3590
GSE80681	Mus musculus	hippocampus	*Klf4*	-1.2879	0.0015
GSE80681	Mus musculus	hippocampus	*Foxj2*	-1.6020	0.0002
GSE60820	Mus musculus	cerebral cortex	*Csrnp1*	1.2500	0.0002
GSE104381	Mus musculus	cerebral cortex	*Mxi1*	-1.9070	0.0010
GSE97537	Mus musculus	hemisphere	*Csrnp1*	1.1000	0.0006
GSE202659	Mus musculus	hemisphere	*Csrnp1*	1.4324	0.0300
GSE202659	Mus musculus	hemisphere	*Nkx6-1*	-2.5850	0.1100
GSE173544	Mus musculus	whole brain	*Zfp523*	-1.4054	0.0001
GSE173714	Mus musculus	whole brain	*Mxi1*	-1.2364	0.0002
GSE131193	Mus musculus	brain microvessel fragment	*Csrnp1*	1.0615	0.1000
GSE131193	Mus musculus	brain microvessel fragment	*Rxrg*	-1.8678	0.0200
GSE45703	Mus musculus	primary neuron	*Csrnp1*	1.0768	0.0001
GSE163614	Rattus norvegicus	cerebral cortex	*Rxrg*	-1.4307	0.0400
GSE163614	Rattus norvegicus	cerebral cortex	*Nkx6-1*	-1.3421	0.2000
GSE106680	Rattus norvegicus	hemisphere	*Zfp523*	-1.1580	0.0055
GSE106680	Rattus norvegicus	hemisphere	*Nkx6-1*	-1.2387	0.2970
GSE106680	Rattus norvegicus	hemisphere	*Nfatc4*	-2.4026	0.0004
GSE106680	Rattus norvegicus	hemisphere	*Zfp523*	1.1580	0.0056
GSE21136	Rattus norvegicus	blood	*Mxi1*	-1.7453	0.1559
GSE21136	Rattus norvegicus	blood	*Klf5*	-1.3442	0.0220

Supplementary Table 7. Comparison of key genes with published GEO datasets in VaD-related diseases.

GSE number	Sample organism	Sample type	Gene name	Log_2(fold change)	*P* value
GSE157628	Homo sapiens	microsample from middle cerebral artery	*Trip12*	1.2600	0.0811
GSE157628	Homo sapiens	microsample from middle cerebral artery	*Pxn*	-1.0700	0.0564
GSE186798	Homo sapiens	astrocyte	*Tpm1*	1.0665	0.0564
GSE111782	Homo sapiens	carotid plaque	*Trip12*	1.0672	0.2540
GSE80681	Mus musculus	hippocampus	*Serpine1*	-4.6639	0.0001
GSE134257	Mus musculus	hippocampus	*Serpine1*	-1.1342	0.0190

GSE60820	Mus musculus	cerebral cortex	*Tpm1*	1.4100	0.0001
GSE107983	Mus musculus	primary microglial	*Serpine1*	-1.1900	0.0022
GSE37777	Rattus norvegicus	cerebral cortex	*Col1a1*	-1.3928	0.0860
GSE17929	Rattus norvegicus	whole brain	*Serpine1*	-2.4737	0.1589
GSE106680	Rattus norvegicus	hemisphere	*Pxn*	-1.3935	0.0042
GSE106680	Rattus norvegicus	hemisphere	*Plec*	-1.2728	0.0013
GSE106680	Rattus norvegicus	hemisphere	*Serpine1*	-4.0217	0.0005
GSE106680	Rattus norvegicus	hemisphere	*Col1a1*	-4.7979	0.0001
GSE106680	Rattus norvegicus	hemisphere	*Nedd4l*	1.7561	0.0167
GSE162072	Rattus norvegicus	middle cerebral artery	*Serpine1*	-2.8677	0.0001
GSE21136	Rattus norvegicus	blood	*Nedd4l*	1.1768	0.0200
GSE148841	Rattus norvegicus	blood	*Tpm1*	1.1923	0.0152
GSE148841	Rattus norvegicus	blood	*Plec*	-1.2006	0.0570
